# Integrating the Nano–Phyto–Micro Triad for Climate-Resilient and Sustainable Agriculture

**DOI:** 10.1021/acsomega.5c07151

**Published:** 2025-11-04

**Authors:** Olusola Adeoye Oluwole, Estefânia V. Ramos Campos, Jhones Luiz de Oliveira, Leonardo Fernandes Fraceto

**Affiliations:** † Institute of Science and Technology of Sorocaba, São Paulo State University, Av. Três de Março, 511 - Alto da Boa Vista, 18087-180 Sorocaba, SP, Brazil; ‡ Department of Science Laboratory Technology, Ekiti State University, Ado Ekiti PMB 5363, Nigeria; § B. Nano, Av. Itavuvu, 11.777 - Jardim Santa Cecilia, 18078-005 Sorocaba, SP, Brazil

## Abstract

The convergence of nanotechnology, plant hormones, and plant-associated microbiomes offers a transformative approach for climate-resilient and sustainable agriculture. This perspective examines how nanocarriers can improve the delivery and stability of natural hormones and microbiome-compatible compounds, addressing challenges such as low bioavailability, environmental degradation, and nontargeted effects. Plant hormones, such as auxins, gibberellins, and salicylic acid, play essential roles in stress responses and growth regulation, while beneficial microorganisms contribute to nutrient cycling, pathogen resistance, and resilience against abiotic stresses. However, their practical application remains limited due to their instability and inconsistent performance under field conditions. Recent advancements have demonstrated that innovative nanomaterials, such as polymeric, lipid-based, or silica nanoparticles, can enable the controlled release, targeted delivery, and environmental protection of these compounds. For example, chitosan-based nanoparticles increase root colonization by beneficial microbes and enhance systemic resistance in tomatoes and maize. This review synthesizes the current knowledge and emerging technologies at the nano–phyto–micro interface, highlighting synergistic mechanisms and gaps in regulation, formulation, and large-scale applications. We advocate integrated research strategies that combine omics approaches, advanced formulations, and real-world validations to unlock the full potential of this triad. Aligning nanotechnology with nature-based solutions may pave the way for low-input, high-efficiency farming systems tailored to changing climates.

## Graphical abstract

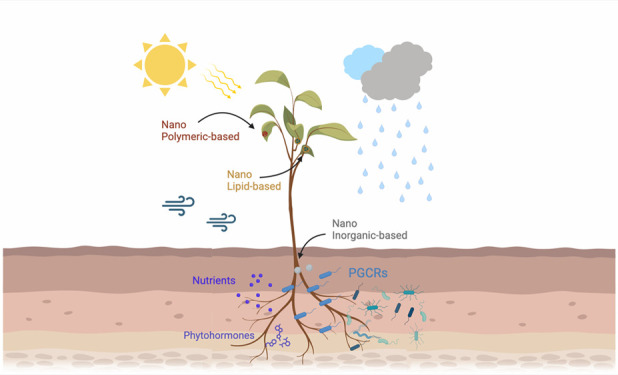

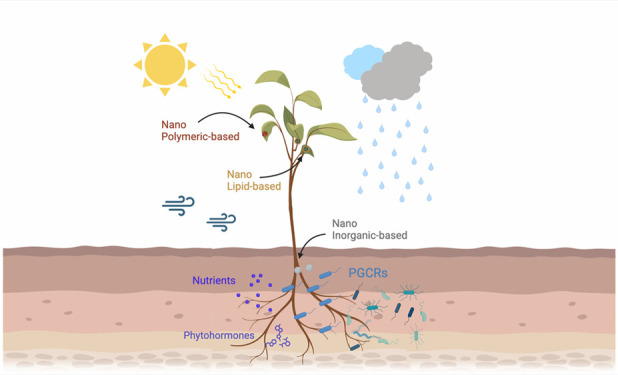

## 1 Introduction

The global population is projected to reach 9 billion by 2050, leading to a significant increase in food demand.^1^ However, the availability of arable land is diminishing, and traditional agricultural practices are struggling to meet this growing need.^2,3^ Food security has gradually become an urgent concern worldwide, particularly in developing countries. According to the Food and Agriculture Organization (FAO), in 2023, approximately 733 million people remain undernourished globally due to food insecurity and malnutrition despite desperate efforts and progress in specific areas, such as stunting and exclusive breastfeeding. An alarming number of people are undernourished as global hunger levels have plateaued for 3 consecutive years. This paradox of feeding a growing global population while ensuring equitable access to food is compounded by climate change-induced disruptions such as droughts, floods, and changing growing seasons, which threaten crop yields and food stability.^4,5^

In addition, the current agricultural model, based on industrial-scale monoculture and intensive farming, has proven to be unsustainable, contributing to environmental damage, such as soil erosion, loss of biodiversity, and excessive water use.^6^ Intensive farming practices often lead to soil degradation, reduced soil fertility, and water contamination due to excess fertilizers and pesticides.^7^ Furthermore, agriculture is a major contributor to greenhouse gas emissions, primarily from livestock production and fertilizer use. Thus, there is an urgent need to transition toward sustainable farming practices that minimize environmental impacts while maintaining high productivity.^8^ Solutions, such as agroecology, organic farming, and integrated pest management, are being explored; however, they require widespread adoption, policy support, and technological innovation.

Nanotechnology is a rapidly evolving field that offers a revolutionary approach to addressing these pressing challenges.^1,3,9^ Nanotechnology has immense potential to enhance agricultural productivity and sustainability^10^ and has the potential to rejuvenate agriculture. Micro/nanomaterials offer great potential for developing precision farming techniques, enabling farmers to optimize water, fertilizer, and pesticide use.^1,3^ Traditional fertilizers are often inefficient, and a significant portion is lost to the environment. Nanoenabled fertilizers, however, help in the controlled release of nutrients, thus reducing waste and minimizing environmental contamination. This boosts the crop yield and reduces the need for chemical inputs.^11^

Encapsulation technology, initially used in biotechnology for cell and enzyme separation, has significantly expanded. Recently, agriculture has been revolutionized by offering a sustainable alternative to hazardous agrochemicals. This involves the encapsulation of chemicals, nutrients, and seeds within biocompatible materials, triggering their release via external stimuli. This controlled-release method represents a major advancement over conventional spraying techniques, enhancing efficiency, reducing environmental impacts, and improving crop yield.^12^ Capsules can be made in nano (<1.0 μm) or micrometric (1.0–1000 μm) sizes with different membrane materials. Polymeric encapsulation, which leverages both natural (e.g., chitosan and alginate) and synthetic (e.g., poly­(vinyl alcohol)) polymers, offers a controlled-release system for agrochemicals and nutrients. This technology optimizes resource utilization, minimizes environmental impacts by reducing leaching and nontarget exposure, and enhances the efficacy of agricultural inputs.^13^ Similarly, nanopesticides are more efficient than traditional formulations, with lower dosages required to achieve the same effect, which helps reduce environmental pollution and resistance development in pests.^14^ They can also target specific pests without affecting beneficial insects and can be designed to release active ingredients only when needed, thereby improving their effectiveness.^15^

Nanoparticles can be used to enhance the delivery of water and nutrients to plants under stressful conditions, thereby improving their ability to survive and thrive.^16^ Nanoencapsulation techniques protect and deliver growth hormones to plants, enhancing their resilience to stress by maintaining a proper hormonal balance.^17^ Nanosensors can detect the early signs of plant diseases and pests, allowing targeted and timely intervention and improved crop yields.^18^ They can also monitor soil health and detect contaminants, enabling precise, data-driven interventions to optimize agricultural practices.^19^ Nanomaterials, such as carbon nanotubes, can improve the physical properties of soils by increasing their ability to retain water and nutrients, thus making crops more resilient to droughts.^20^

Table 1 summarizes the primary roles and synergistic interactions between nanomaterials, phytohormones, and plant-associated microbiomes in agricultural systems. Each component contributes to plant growth, stress tolerance, nutrient efficiency, and pathogen resistance. The rightmost column highlights the synergistic outcomes when these three domains are integrated, reinforcing a systems-level approach for sustainable crop management and resilience under abiotic and biotic stresses.

**Table 1:** Overview of Synergistic Effects and Functional Contributions of Nanomaterials, Phytohormones, and Microbiomes in Agriculture

Aspect	Traditional Agriculture	Nanoenhanced Agriculture
Input	Manual labor, organic fertilizers	Nanofertilizers and nanopesticides
Sustainability	Environmentally sustainable if practiced properly	Potentially more sustainable due to efficient resource use
Yield	Lower yield and susceptibility to external factors	Higher yields, more resilient to biotic and abiotic stress
Biodiversity	High biodiversity due to polyculture and crop rotation	Can enhance biodiversity through nanopesticides and precision farming
Environmental Impact	Lower impact, though can be affected by soil erosion and pests	Reduced impact through controlled release and minimal pesticide use
Resource Efficiency	Limited efficiency in water and nutrient use	Enhanced water and nutrient use efficiency through nanosensors and nanomaterials

## 2 Symbiotic Interactions between Microbiomes and Plants

Plants and microbes form one of the most complex and diverse symbiotic relationships, through which health and productivity are enhanced in various ecosystems. Emerging findings highlight the role of microbial interventions in not only tailoring the root system but also inducing resistance to environmental stresses, which benefits the uptake of water and nutrients.^21,22^ Symbiotic associations are the physiological unions of microorganisms—bacteria, fungi, algae, and viruses with higher plants—and play critical roles in maintaining ecological sustainability and inducing increased plant growth and development. Figure 1 illustrates the groups of beneficial microbial communities that significantly contribute to the management of plant stress physiology and help resist environmental stresses, including heavy metals, drought, and salinity. They achieve this by transfiguring physiological responses that mediate mechanisms such as stress hormone production, modification of root architecture, and changes in internal signaling.^23^

**Figure 1:**
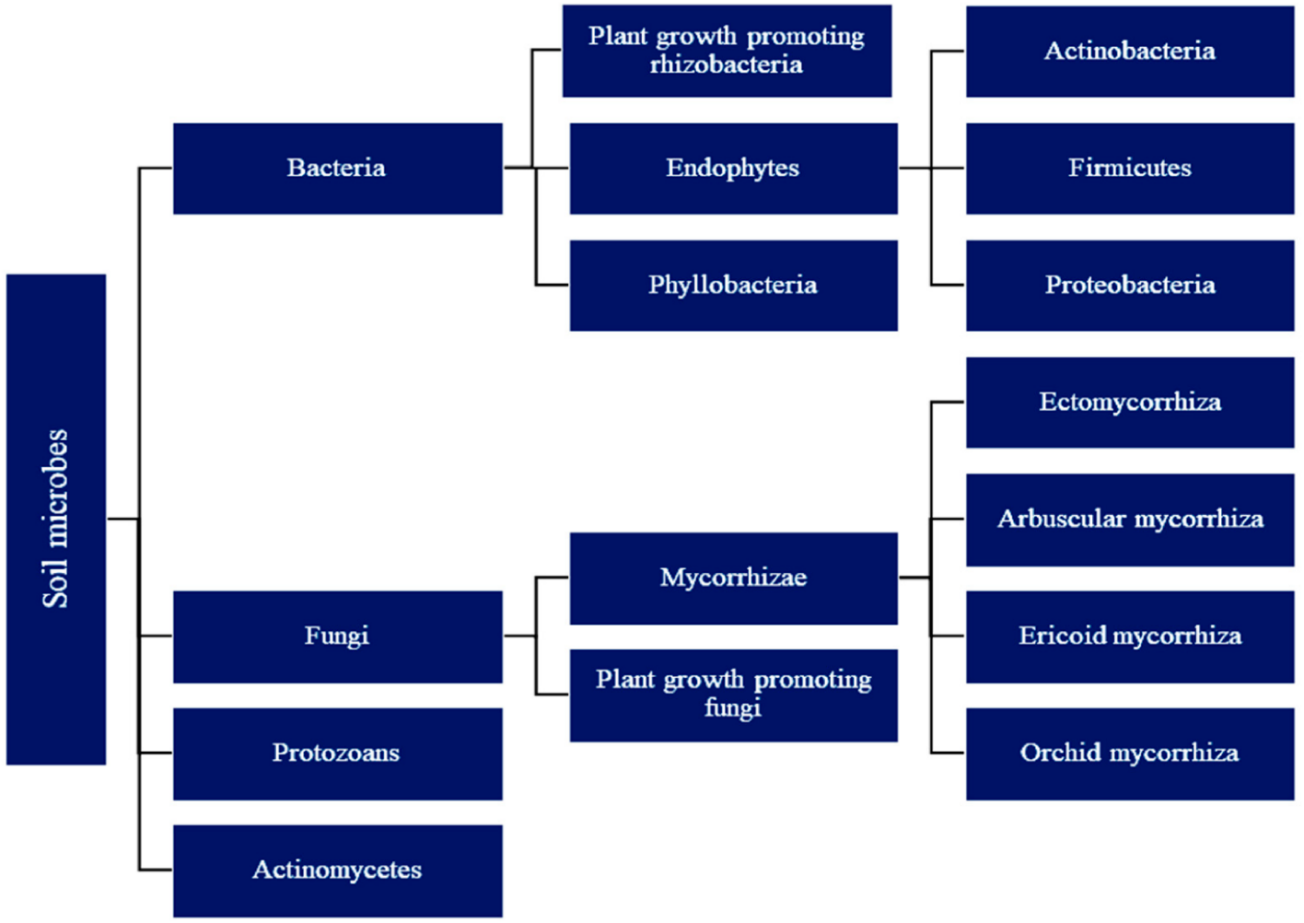
Major groups of soil microorganisms and their functional roles in promoting plant growth and resilience. Bacteria are categorized into rhizobacteria, endophytes, and phyllobacteria, with further subdivisions based on phylogeny (e.g., Actinobacteria, Firmicutes, Proteobacteria). Fungi include plant-growth-promoting fungi and mycorrhizal types (e.g., ectomycorrhiza, arbuscular, ericoid, orchid mycorrhiza). Additional groups, such as protozoans and actinomycetes, also contribute to soil health and nutrient cycling. These microbial communities form a foundational component of the plant microbiome, playing critical roles in nutrient acquisition, stress tolerance, and enhanced agricultural productivity, particularly when integrated with nanobiotechnology and bioactive compounds in sustainable agriculture strategies. Reprinted with permission from Chauhan, P., Sharma, N., Tapwal, A., Kumar, A., Verma, G. S., Meena, M., Seth, C. S., & Swapnil, P. (2023). Soil Microbiome: Diversity, Benefits and Interactions with Plants. *Sustainability*, 15(19), 14643. Copyright 2023/Multidisciplinary Digital Publishing Institute—MDPI.

A significant aspect of their influence is the mobilization and solubilization of nutrients; rhizosphere microorganisms transform nutrients into bioavailable forms. For example, specific mycorrhizal fungi make phosphorus soluble, whereas some bacterial taxa convert insoluble micronutrients into soluble forms for the plants.^24,25^ In addition, arbuscular mycorrhizal fungi enhance photosynthetic efficiency and improve soil structure, thereby substantially increasing nutrient absorption and resilience to various environmental stresses, and contributing to long-term ecosystem stability and plant productivity.^26,27^ This microbial help is meaningful in soils with low nutrient availability and contributes significantly to improving plant growth and yield potential.^28^ In addition, bacterial populations synthesize growth-promoting hormones that have far-reaching consequences on their developmental processes.^29^ Actinomycetes also contribute significantly to the production of bioactive compounds used in biocontrol strategies and improve soil health through the decomposition of organic materials.^30,31^

Microbial communities also affect the physiology of host plants by utilizing phytohormones, such as auxins, cytokinins, and gibberellins, which are crucial for various growth and developmental processes.^32,33^ These microorganisms trigger enhanced defenses against pathogens, thereby improving the overall defense mechanisms of plants.^34^ Chemical and enzymatic interactions significantly contribute to the foundation of plant–microbe relationships. While microbes enhance nutrient availability and detoxify harmful substances, plants provide nutrients and signaling molecules for microbial growth and activity.^35^ Research on plant–microbe interactions can be summarized into two themes: plant responses to microbe-induced changes and the stimulation of or resistance by a given plant toward microbial activities. The first theme includes induced systemic resistance (ISR), which defines the local and systemic defenses enabled by microbial interactions.^36^

Furthermore, it has been demonstrated that microbial communities can alter the balance of phytohormones in plants, influencing essential growth parameters such as root and shoot formation, flowering, and senescence.^37,38^ Therefore, multiple modes of interaction between plants and microbes are critical for plant adaptation to various environmental conditions and stressors.^39,40^ Figure 2 and Table 2 show the mechanisms by which beneficial soil microbiomes help plants manage soil stress.

**Table 2:** Summary of the Key Interactions between Different Soil Microbiome Groups and Plants for Sustainable Agriculture

Organism Group	Key Roles	Examples	Mechanistic Insights
**Bacteria**	**a. Nutrient Cycling & Acquisition:** Fix atmospheric nitrogen (Biological Nitrogen fixation), phosphate solubilization and produce iron-chelating siderophores.	*Rhizobium* (BNF with legumes),	Chemical communication via root exudates, siderophores, and quorum sensing. Production of signaling molecules (e.g., Nod factors).
		*Pseudomonas* sp. (nutrient solubilization,	
		IAA phytohormone production by *Bacillus subtilis* and *Azospirillum*.	
		ISR by *Bacillus subtilis*	
	**b. Phytohormone Production:** Synthesize and secrete growth regulators e.g. auxins, gibberellins, and cytokinins.		
	**c. Pathogen Protection:** Produce antibiotics (antibiosis), compete for resources, and trigger Induced Systemic Resistance (ISR).		
	**d. Stress Tolerance:** Produce ACC deaminase to lower stress ethylene and form biofilms to improve soil moisture.		
**Fungi**	PGPF are non-pathogenic saprotrophic fungi which are either endophytic or epiphytic		Chemical communication via signaling molecules (Myc factors). Mycorrhizal networks facilitate inter-plant nutrient and signal transfer
	**a.Nutrient & Water Uptake:** Form mycorrhizal symbioses (AMF, Ectomycorrhizal) to extend the root’s absorptive surface area, accessing immobile nutrients like phosphorus.		
	**b.** improve seed germination and seedling vigor in different agronomic and horticultural crops	*T. harzianum* increased seed germination, emergence index, seedling vigor and successful transplantation percentage in muskmelon compared to the untreated controls	
	**c. Biocontrol**	*Trichoderma, Sphaerodes mycoparasitica*), produce antifungal compounds, and trigger ISR.	
	**d .Stress Tolerance:** Produce osmolytes and antioxidants to mitigate stress.	a. Increased tolerance to salt and oxidative stress by *T. harzianum* T-2	
		b. Enhanced copper stress tolerance in maize seedling by *Chaetomium globosum*	
		c. Increased tolerance to drought stress *T. atroviride* ID20G	
	**e .Growth Promotion:** Synthesize and secrete phytohormones.	a .Auxin-related compounds (indole-3-acetic acid, IAA) by *T. virens* Gv. 29-8	
		*b.*Gibberellins (production by *A. fumigatus* HK-5-2 and *Penicillium resedanum* LK6	
**Algae** *(Includes cyanobacteria)*	**a. Biofertilization:** Fix atmospheric nitrogen and provide it to plants.	*Nostoc*, *Anabaena* (nitrogen fixation), *Chlorella* (antioxidants), various microalgae.	Chemical signaling through phytohormones and biostimulant compounds. Can prime plant immunity.
	**b.Biostimulation:** Produce phytohormones (auxins, cytokinins) and other bioactive compounds that enhance plant metabolism and growth.		
	**c.Biocontrol:** Produce compounds with antifungal and antibacterial activity.		
	**d.Stress Tolerance:** Increase plant antioxidant activity and aid in heavy metal detoxification.		
**Protozoa**	**a.Nutrient Mineralization:** Graze on soil bacteria, stimulating nutrient turnover in the “microbial loop” and releasing nutrients like nitrogen and phosphorus for plant uptake.	Amoebae, flagellates, ciliates (common soil protozoa).	Selective grazing alters microbial community dynamics. Interactions can favor specific bacterial strains that produce phytohormones.
	**b .Bacterial Community Modulation:** Alter the composition of the soil microbiome through selective predation, potentially favoring beneficial bacteria.		
	**c .Root Architecture Influence:** Impact root morphology indirectly by influencing phytohormone-producing bacteria.		

**Figure 2:**
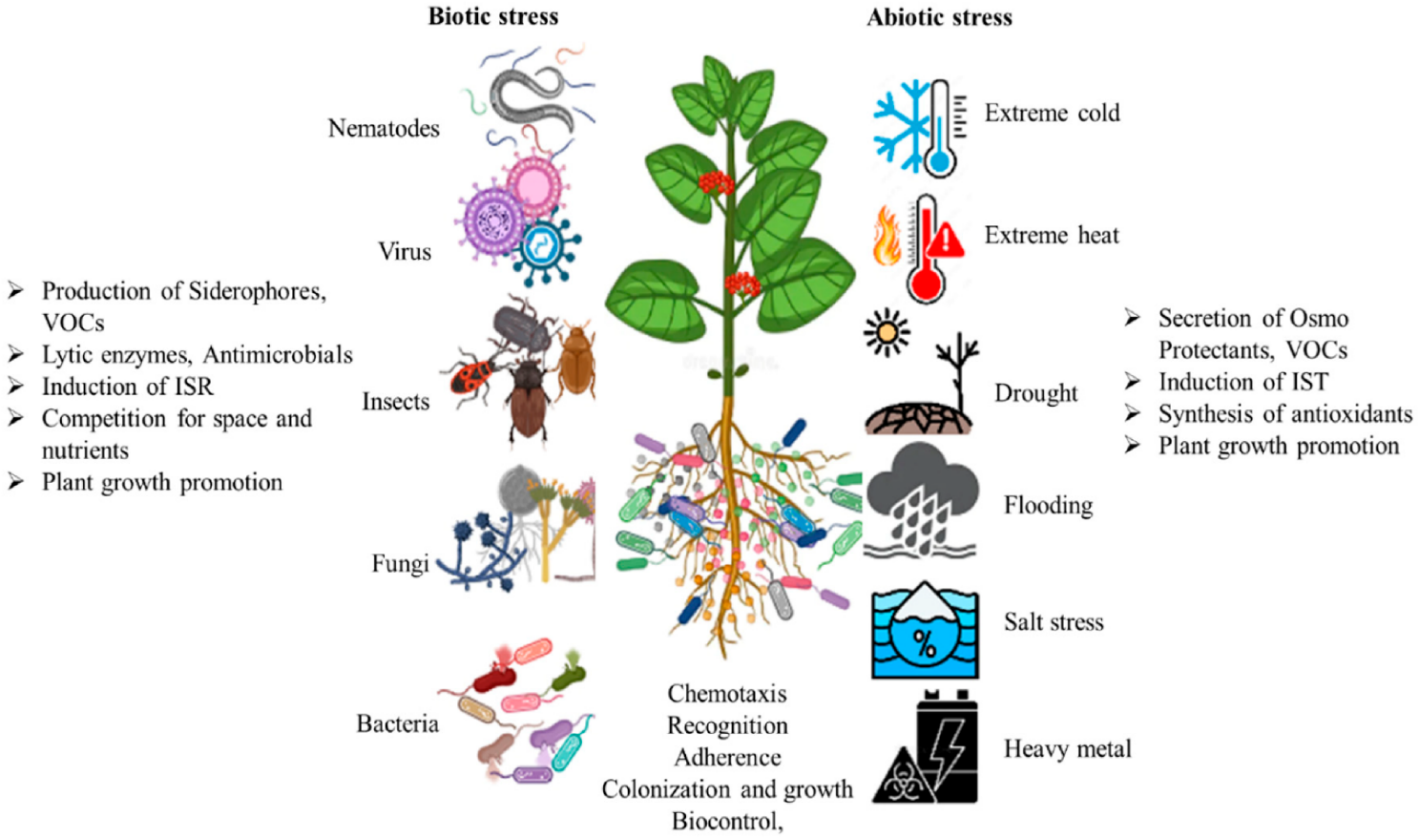
Microbial-mediated mechanisms of plant tolerance to biotic and abiotic stressors. This illustration depicts how beneficial microorganisms contribute to plant resilience under various environmental conditions. Listed on the left are microbial actions that mitigate biotic stress factors such as nematodes, viruses, insects, fungi, and bacteria through mechanisms including siderophore and volatile organic compound (VOC) production, secretion of antimicrobial enzymes, activation of induced systemic resistance (ISR), and competitive exclusion. Listed on the right are mechanisms by which microorganisms support plant defense against abiotic stresses such as extreme temperatures, drought, flooding, salinity, and heavy metal toxicity by producing osmoprotectants, antioxidants, and VOCs, enhancing plant growth. In the root zone, microbes interact through chemotaxis, recognition, adherence, colonization, and biocontrol, forming a dynamic rhizosphere interface that enhances overall plant tolerance and productivity. Reprinted with permission from Tharanath, A. C., Upendra, R. S., & Rajendra, K. (2024). Soil Symphony: A Comprehensive Overview of Plant–Microbe Interactions in Agricultural Systems. *Applied Microbiology*, 4(4), 1549–1567. Copyright 2025/Multidisciplinary Digital Publishing Institute—MDPI.

The plant microbiome is a dynamic ecosystem in which bacteria, fungi, algae, and protozoa interact in a complex network that has a dramatic impact on plant health and productivity. These microorganisms play critical roles in nutrient cycling and acquisition, as well as pathogen suppression and stress tolerance, which are mediated by all four groups. These connections are mediated by a complex chemical conversation that includes root exudates and microbial signaling molecules, as well as cooperative behaviors such as biofilm formation and sophisticated symbiotic signaling pathways. Agriculture may shift toward more sustainable and resilient practices by understanding and leveraging these complex relationships, notably through the application of modern biotechnologies.

## 3 Current State and Advancements in the Use of Nanomaterials in Agricultural Systems

Nanomaterials are emerging as vital tools for enhancing agricultural productivity and soil health, with approximately 1300 commercial formulations utilizing their unique properties.^41–43^ Notable advancements include the use of nanofertilizers, which significantly improve nutrient use efficiency and yield, as demonstrated by a 32% growth rate and a 20% seed yield increase in soybeans.^20,44^ This approach supports sustainability by minimizing nutrient loss.^45^ Furthermore, nanopesticides and nanobiosensors have transformed pest management and agricultural monitoring.^46,47^ Nanopesticides enhance the effectiveness of pest control while reducing toxicity to nontarget organisms, although health risk assessments are important.^48–51^

Nanobiosensors offer improved sensitivity for the early detection of soil and plant health indicators.^52–54^ Despite their benefits, challenges such as high development costs may limit their broader adoption in commercial agriculture.^55,56^ Figure 3 summarizes the current applications of nanotechnology in agriculture.

**Figure 3:**
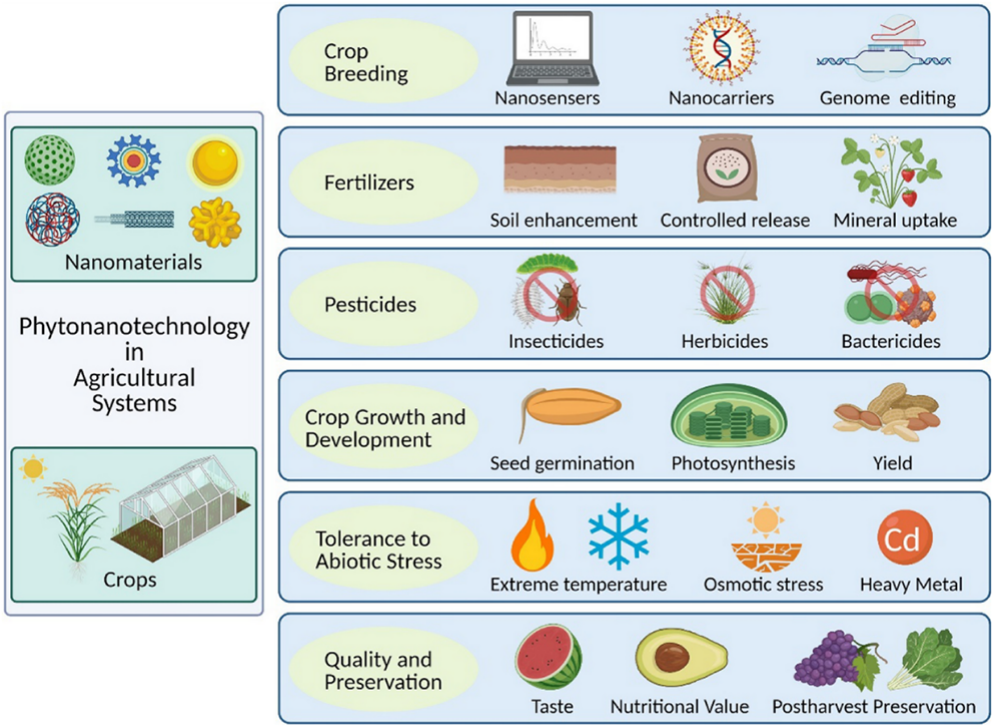
Schematic representation of nanotechnology applications in agricultural systems. This schematic illustrates the diverse roles of nanotechnology across various stages of agricultural production. It includes the precision delivery of agrochemicals (e.g., pesticides, herbicides, and fertilizers), nanosensors for real-time monitoring of plant health and soil conditions, nanoparticle-mediated stress tolerance, and enhanced nutrient uptake. The diagram emphasizes how nanotechnology enables targeted action, reduces environmental impact, and contributes to sustainable and efficient crop management by integrating with biological and environmental signals. Reprinted (Adapted) with permission from Jiang, M., Song, Y., Kanwar, M. K., Ahammed, G. J., Shao, S., & Zhou, J. (2021). Phytonanotechnology applications in modern agriculture. *Journal of Nanobiotechnology*, 19, 1–20. Copyright 2021/BMC-Springer Nature.

### 3.1 Designing Delivery Systems for Responsive Nutrient Release in Agriculture

The development of innovative delivery systems for fertilizers and nutrients that respond to specific plant or environmental cues is crucial for enhancing agricultural sustainability and efficiency. This approach minimizes nutrient waste while maximizing plant uptake, reflecting advancements in materials science, plant physiology, and soil chemistry. Stimuli-responsive polymers offer a dynamic approach to nutrient delivery and are designed to react to environmental changes such as pH, temperature, and moisture (Table 3). The chemical and physical properties of these materials can be altered after exposure to external stimuli.^57^ This versatility allows the tailoring of specific mechanical, chemical, electrical, optical, biological, or other properties. Such systems can be engineered in various forms, including bulk materials, thin films, micro/nanoparticles, and composites. The formulation of stimuli-sensitive carriers is an effective strategy for improving controlled release systems, in which macromolecular carriers respond to subtle environmental signals and modify their physicochemical properties to facilitate the release of encapsulated substances.^58,59^ In agrochemical applications, these carriers enable the targeted release of active compounds in response to abiotic and biotic stimuli, including light, pH, temperature, magnetic fields, and enzymes.^60^

**Table 3:** Responsive Nutrient Delivery Systems for Sustainable Agriculture

Delivery System Type	Nutrients Delivered	Responsive Trigger(s)	Release Mechanism	Key Materials and Technologies	Citation
**Coated Nanoparticles**	Nitrogen (urea), Macronutrients (NPK), Micronutrients (Fe, Zn)	pH, temperature, microbial activity	Coating degradation, Diffusion	**Coating:** Chitosan, starch, zein, cellulose, ethyl cellulose, polylactic acid (PLA), sulfur, lignin, polyurethane. **Core:** Nutrients encapsulated within the particle. **Examples:** Starch-based latex reinforced with char for NPK release, polyurethane-coated urea for sustained N release.	Kekeli et al.^70^
**Hydrogels**	Macronutrients (NPK), Nitrogen (urea), Micronutrients (Cu, Zn, P)	Swelling (moisture, ionic strength), Biodegradation (enzymes, microbes), pH, Temperature	Controlled swelling and gradual breakdown of the polymer matrix	**Hydrogel Matrices:** Natural biopolymers like cellulose, alginate, starch, and chitosan; synthetic polymers like poly(acrylic acid). **Cross-linking Agents:** Epichlorohydrin, glutaraldehyde, or ionic cross-linkers. **Examples:** Starch-based hydrogels that release urea based on water absorption; cellulose-based hydrogels that release nutrients in a pH-dependent manner.	Mikhailidi et al.^71^
**Metal–Organic Frameworks (MOFs)**	Micronutrients (Zn, Fe), Macronutrients (N, P)	pH, Reduction (redox-responsive)	Framework degradation or controlled release through porous channels	**Metal Ions:** Iron (Fe), Zinc (Zn). **Ligands:** Organic ligands like 2-methylimidazole. **Examples:** Fe-MOFs that release N, P, and Fe in a controlled pattern or zeolitic imidazolate framework-8 (ZIF-8) that releases Zn under acidic conditions.	Wu et al.^72^
**Micelles**	Micronutrients, especially lipophilic nutrients	Redox, pH, enzymes	Aggregation/disaggregation of the amphiphilic micelle structure	**Amphiphilic Polymers:** Biodegradable polymers with hydrophilic and hydrophobic sections like chitosan derivatives. **Payload:** Nutrients encapsulated within the hydrophobic core. **Example:** Polymeric micelles that release nutrients in response to reactive oxygen species (ROS) or specific enzymes.	Janik et al.^72b^
**Nanofibers**	Macronutrients (NPK), Micronutrients (Fe, Zn), Water	Biodegradation, swelling	Degradation of the fibrous matrix by soil microbes and moisture; controlled diffusion	**Fiber Materials:** Poly(vinyl alcohol) (PVA), cellulose, polylactic acid (PLA). **Fabrication:** Electrospinning. **Examples:** PVA nanofibers loaded with multinutrients that decompose and release nutrients slowly when coated on seeds.	Mohanraj *et al.* ^73^
**Nanocomposites and Hybrid Materials**	Macronutrients (NPK), Micronutrients (Cu, Zn, Mn)	Dissolution, ion exchange, pH, humidity	Gradual dissolution, ion exchange, and controlled release from the composite matrix	**Components:** Nano hydroxyapatite (nHAP), nano urea, graphene oxide (GO), and micronutrients (Cu, Zn, Mn). **Mechanism:** The nHAP acts as a carrier, releasing nutrients upon dissolution; GO can hold and release micronutrients.	Haydar et al.^74^

Stimuli-responsive delivery systems can utilize natural polymers such as alginate, chitosan, and ethyl cellulose, as well as silica materials, owing to their availability and biodegradability.^61^ Recent studies have also explored pesticide and herbicide delivery carriers, highlighting their responsiveness to environmental stimuli.^62^ The application of stimuli-sensitive carriers in nanoformulations facilitates the controlled release of various compounds in a highly effective manner at specific times. This approach enhances bioefficacy while minimizing side effects, reducing the required dosage, and decreasing the frequency of application. Stimuli-responsive particles represent a promising yet nascent technology that requires further exploration to broaden its application in agroindustry.^61^

The integration of plant signals into nutrient delivery systems enhances their efficacy. Plant-based biosensors utilizing living plants or plant materials offer cost-effective and noninvasive solutions for environmental monitoring and food safety.^63^ Biomimicry and bioinspired coatings have shown significant potential for enhancing nutrient uptake and translocation. For instance, plant viruses utilize passive transport along with photoassimilates to migrate over extended distances within infected plants. Using specific cell receptors, coating nanoparticles (NCs) with biorecognition patterns (such as sugars or peptides) may promote their absorption and translocation in plants.^63,64^

Carbon dot (CD)-based NCs coated with sucrose or guiding peptides improve the transport of chemical payloads into the phloem or chloroplasts, creating carriers based on plant viruses for better nematicide delivery to plants through the soil.^65,66^ Mesias and Penaloza^67^ designed a carboxymethylcellulose-based encapsulant system for the controlled release of nitrogen–phosphorus–potassium (NPK) fertilizer using alginate as a stabilizer and citric acid as a cross-linking agent. They observed that under various pH conditions, the system conformed to the standards of controlled-release fertilizers, with a maximum release rate of 50% over 30 days. In addition, Su et al.^68^ confirmed that deep application of controlled-release urea (CRU) could enhance both the yield and quality of *P. notoginseng* while reducing the amount of applied fertilizer.

Kubavat et al.^69^ introduced slow-release chitosan nanoparticles (CNK), which demonstrated remarkable results in boosting the fresh and dry biomass of *Zea mays* by 51% and 47%, respectively, when utilizing a reduced potassium rate (75% CNK). This approach outperformed traditional treatments using 100% KCl, improved soil physical properties, doubled root biomass, and enhanced microbial activity and carbon cycling. Some of these delivery systems that have been applied in agriculture are summarized in Table 3 below. The development of innovative delivery systems for fertilizers and nutrients that respond to specific plant or environmental cues is crucial for enhancing agricultural sustainability and efficiency. By leveraging stimuli-responsive polymers and targeted delivery systems based on plant signals, nutrient waste can be minimized while maximizing plant uptake, ultimately leading to more sustainable agricultural practices.

### 3.2 Targeted Delivery of Phytohormones

Recent advancements in nanotechnology have transformed agriculture by introducing innovative strategies to enhance crop yield and sustainability. The precise delivery of phytohormones via nanoparticles offers significant potential for optimizing plant growth, stress tolerance, and resource utilization. Phytohormones, such as auxins, gibberellins, cytokinins, abscisic acid, and ethylene, are essential for coordinating various physiological processes in plants. Nonetheless, application methods such as soil amendments or foliar sprays are often compromised by rapid degradation, inefficient distribution, and suboptimal absorption. Consequently, higher doses are required, increasing costs and environmental risks. Harnessing nanotechnology addresses these limitations by enabling the controlled release, targeted delivery, and enhanced availability of phytohormones. Nanoparticles protect against ecological degradation, while facilitating precise transfer to specific plant tissues or cells. Various nanocarriers, including biodegradable polymers (such as chitosan and polylactic acid), liposomes, and inorganic nanomaterials, have shown efficacy in encapsulating and prolonging phytohormone release (Table 4).

**Table 4:** Targeted Delivery of Phytohormones Using Nanotechnology

Phytohormone(s)	Nanoparticle/Polymer Carrier	Encapsulation Technique	Target Plant/Application	Effect
**Auxin (e.g., IAA, IBA)**	• Chitosan nanoparticles (NPs)	• Ionic gelation	• *Malus pumila* (apple) roots	• Protected auxin from degradation, providing slow, controlled release and enhancing rooting efficiency
Butova et al.^76^	• Silver NPs	• Thermal reduction	• Crop yield enhancement	
	• Organic formulations from olive residues	• Lipid extraction		
				• Improved bioavailability and efficacy of auxins for better yields
Hanif et al.^77^	• Copper oxide (CuO) nanomaterials	N/A	• Chickpea plants	• Enhanced physiological parameters, including root length, shoot length, and biomass
**Gibberellic Acid (GA3)**	• Chitosan NPs	• Encapsulation in polymer matrix	• *Phaseolus vulgaris* (common bean)	• Significantly increased leaf area and levels of chlorophylls and carotenoids
Santo Pereira et al.^78^	• Alginate/chitosan nanocarriers	• Nanoprecipitation	• *Solanum lycopersicum* (tomato)	
				• Enhanced overall plant development and fruit productivity
**Abscisic Acid (ABA)** Valdes et al.^79^	• Various NPs (interacts with and affects signaling)	N/A	• *Arabidopsis thaliana* (as a stress-response hormone)	• Nanoparticles have been shown to influence ABA signaling pathways, affecting stress tolerance and stomatal closure
**Cytokinin (CK)**	•Chitosan nanoparticles	Encapsulation within polymer matrix	*Arabidopsis thaliana* *Capsicum annum*	• Nanoparticles influence cytokinin signaling, affecting processes like cell division and delaying senescence
Vinković et al.;^80^ Soni et al.^81^	• Engineered nanomaterials like quantum dots and carbon-based nanoparticles	Ion gelation		
	• Silver NPs			Also influences plant growth and stress resilience
**Jasmonic Acid (JA)/Methyl Jasmonate (MeJA)**	• Chitosan NPs	•Encapsulation	• *Oryza sativa* (rice) (JA)	• Prolonged the elicitation of phenolic and flavonoid compounds in plants Delayed the ripening process and maintained the quality of cherry tomatoes during storage
Giménez-Bañón et al.;^82^ Arya et al.^83^	• Carbon nanotubes, ferromagnetic alloys	•Evaporative loading techniques	*•* *Camellia sinensis*	
			• *Vitis vinifera*	
			*•* *Solanum lycopersicum*	Protected MeJA from degradation and provided sustained release on leaves, ensuring activity for longer periods
**Salicylic Acid (SA)**	• Mesoporous silica NPs coated with chitosan (MSN-chitosan)	Ionic gelation	• Rice plants (for cadmium tolerance)	• Controlled release of melatonin, improved photosynthesis, enhanced antioxidant activity
Polyakov et al.^84^	• Nanoscale hydroxyapatite (nHA)		• Tomato plants (fungal wilt)	
				• Increased antioxidant compounds and improved disease resistance
**Melatonin (MT)** Chen et al.^85^	• Mesoporous silica NPs coated with chitosan (MSN-chitosan)	Ionic gelation	• Rice plants (for cadmium tolerance)	• Controlled release of melatonin, improved photosynthesis, enhanced antioxidant activity, and gene expression level regulation

Moreover, surface functionalization with tailored ligands enables directed delivery, thereby improving uptake efficiency and minimizing off-target interactions that may interfere with plant physiology.^75^ This nanotechnology-based approach is promising for combating abiotic stressors such as drought, salinity, and heat. For example, abscisic acid-loaded nanoparticles have been shown to promote water-use efficiency by regulating stomatal activity under drought stress. Similarly, auxin-loaded nanocarriers boost root development under saline conditions by supplying plant hormones exclusively to the root system for maximum benefit.^77^ Table 4 briefly summarizes the targeted delivery of key phytohormones that are crucial to plant health and development using nanomaterials. However, despite their remarkable potential, large-scale deployment faces hurdles such as uncertainty regarding nanoparticle toxicity, environmental persistence, production scalability, and cost-effectiveness. Addressing these concerns requires comprehensive research and development of environmentally friendly and economically viable approaches. In conclusion, nanoparticle-mediated delivery of phytohormones offers a transformative solution for precision agriculture by enhancing crop performance, increasing efficiency, and reducing the impact of waste. Advancing this innovation is vital for driving sustainable agricultural practices globally.^42^

### 3.3 Emerging Technologies for Encapsulation and Controlled Release

Nanotechnology-based systems are at the forefront, offering precise control over the delivery of phytohormones. Biodegradable nanoparticles, nanogels, and nanoemulsions are commonly used for encapsulation, protection against environmental degradation, and targeted and sustained release (Table 5). Encapsulation is a highly effective method for entrapping bioactive compounds within polymer materials to safeguard and deliver them at an appropriate time and target location.^86^ This approach creates particles with enhanced hydrophilicity and lipophilicity, which improve their ability to penetrate plant tissues. This process involves enclosing a bioactive compound (core material) within a protective carrier to form capsules with improved biological characteristics. The coating material creates a matrix that safeguards the core from environmental factors such as heat, oxygen, light, pH, and shear, thereby inhibiting volatilization and reducing degradation sensitivity. Encapsulation effectively addresses the physical and chemical instabilities of phytohormones (PHs).^87,88^

**Table 5:** Emerging Encapsulation and Controlled-Release Technologies in Agriculture

Types of Delivery System Materials	Types of carrier nanomaterialCNs	Advantages over Conventional Methods	Mechanism of Delivery
Inorganic Nanocarriers	• Mesoporous silica nanoparticles (MSNs)	• Encapsulating macro- and micronutrients	• Targeted Release: Can be functionalized to release nutrients in specific soil pH or moisture conditions.
	• Clays (e.g., zeolite, bentonite) and layered double hydroxides (LDHs)	• Soil amendment and water retention	• Improved Efficiency: Nanoscale size improves solubility, dispersion, and nutrient uptake.
	• Biochar	• Reducing phosphorus fixation in soil	• Environmental Benefits: Higher nutrient-use efficiency and lower environmental residues.
Organic and Composite Carriers	• Biopolymer hydrogels (e.g., alginate, chitosan, cellulose)	• Sustained, gradual nutrient release	• Programmable Release: Can be designed to match nutrient release with specific crop growth stages.
	• Polymer-coated granules (using natural or synthetic polymers)	• Enhanced water retention in soil	• Reduced Labour and Costs: Less frequent application is needed, reducing labor, energy, and costs.
	• Nanocomposites (combining organic and inorganic components)	• Biodegradable coatings reduce plastic waste	• Increased Yield and Quality: Supports steady growth, leading to higher crop yields and nutritional quality.
Stimuli-Responsive Systems	• Polymers sensitive to pH, temperature, or enzymes	• Releasing nutrients based on environmental cues	• Responsive Delivery: Release of nutrients or pesticides can be triggered by specific biotic or abiotic signals.
	• Light-responsive materials	• Protecting active ingredients from UV degradation	• Higher Efficacy: Targeted application improves the effectiveness of nutrients and pesticides.
	• Redox-responsive nanocarriers	• Target-specific pest control (e.g., nematodes)	• Reduced Waste: Release is optimized to reduce nutrient loss from leaching and volatilization.
Nanostructures	• Nanoparticles (e.g., from chitosan, lignin, zein)	• Encapsulating and delivering nutrients and pesticides	• Enhanced Absorption: Smaller particle size enables better uptake by plants at the cellular level.
	• Micelles and Nanoemulsions	• Foliar sprays with enhanced adhesion	• High Loading Capacity: MOFs and other nanostructures have a large surface area for carrying substances.
	• Nanofibers	• Precision pest management	• Increased Stability: Carriers can protect sensitive nutrients and pesticides from degradation.
	• Metal–Organic Frameworks (MOFs)		

Sampedro-Guerrero et al.^89^ investigated the effects of salicylic acid (SA) on *Arabidopsis thaliana* development and explored the role of encapsulation in mitigating these effects. They discovered that free SA adversely affected root length, growth rate, and gravitropic responses in a dose-dependent manner, primarily due to increased endogenous SA levels, which lowered auxin concentrations. The use of silica and chitosan capsules for SA encapsulation significantly reduced the adverse effects of free SA. This technique allows for controlled release, regulation of hormone uptake, and minimization of damage to plant growth and responses. The schematic in Figure 4 illustrates the integration of advanced biotechnological tools for sustainable crop management. This highlights the use of biopolymer-based nanocarriers, multifunctional platforms for the codelivery of active compounds (e.g., nutrients, pesticides, and bioactives), and stimuli-responsive systems capable of releasing payloads in response to environmental or biological cues. The model also emphasizes the synergistic interactions with plant-associated microbiomes to boost plant health and productivity. Together, these innovations aim to minimize agrochemical inputs, enhance resource use efficiency, and reduce the ecological footprint, paving the way for resilient and environmentally conscious agriculture.

**Figure 4:**
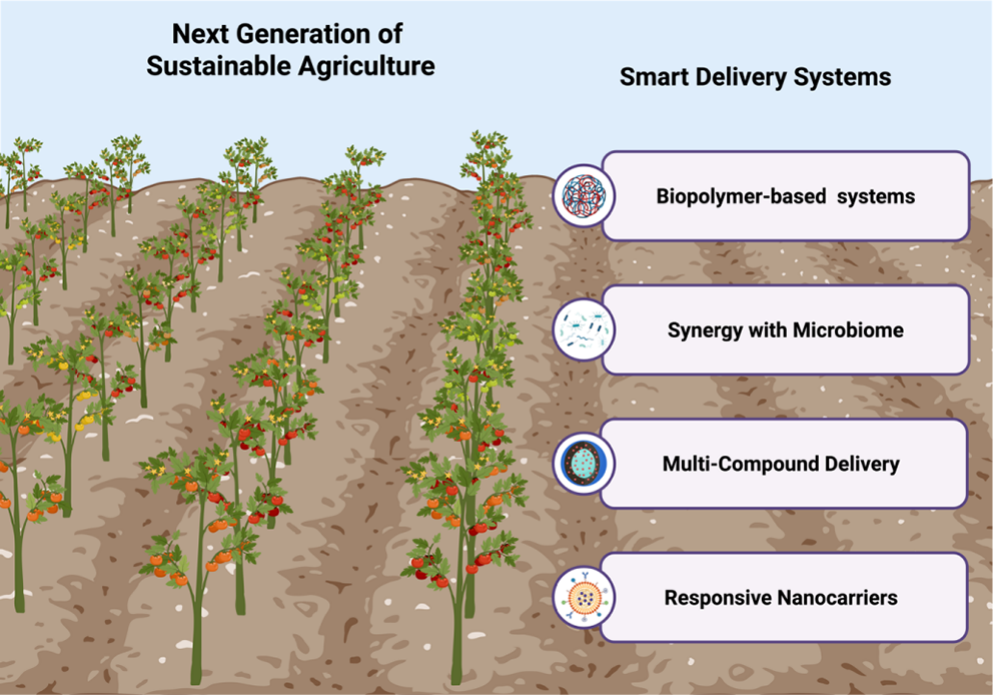
Vision for the next generation of sustainable agriculture through advanced smart delivery systems. This schematic illustrates how emerging innovative delivery technologies are revolutionizing agricultural practices. The left side represents productive, healthy crops sustained by innovative approaches that minimize environmental impact. On the right, four key strategies are highlighted: (i) biopolymer-based systems that allow controlled and biodegradable release of agrochemicals; (ii) integration with plant-associated microbiomes to enhance resilience and nutrient uptake; (iii) multicompound delivery platforms enabling simultaneous transport of bioactives (e.g., nutrients, hormones, and protectants); and (iv) responsive nanocarriers that release their payload in response to specific environmental or biological stimuli. These approaches define a forward-looking framework for regenerative and efficient agricultural systems.

#### 3.3.1 Biopolymer-Based Encapsulation

Biopolymer-based encapsulation is an innovative approach that leverages natural biodegradable materials, such as hydrogels, alginate beads, and chitosan. These carriers enable the gradual release of phytohormones in response to environmental conditions, such as moisture or soil pH. Hydrogels are three-dimensional networks of hydrophilic polymers that absorb large amounts of water, whereas alginate beads are formed from alginic acid. These materials allow the gradual release of phytohormones as they swell and shrink in response to moisture levels, thereby controlling the release rate. They help maintain sustained hormone availability during critical growth phases.^90^ Chitosan is a biodegradable polymer that enhances phytohormone encapsulation. It forms nanoparticles that protect phytohormones from degradation and allow for controlled release. It is particularly effective in delivering hormones to combat plant pathogens and improve their growth.^91^ Caballero et al.^92^ developed a microencapsulation formulation using alginate matrices cross-linked with calcium chloride to encapsulate and achieve the controlled release of *Oscheius carolinensis* nematodes for the biological control of *A. ipsilon* in vegetable crops, with considerable on-field success. Yuan et al.^93^ used a combination of gelatin, chitosan, and polylactic acid to regulate urea release. This composite prolonged the diffusion time of urea by up to 1350 times compared to that of pure urea. Kaur et al.^94^ managed the release kinetics of urea and ammonium dihydrogen phosphate with carboxymethyl cellulose (CMC) that was cross-linked using citric acid. The three-dimensional matrix controls the release of these substances in water for over 40 h, with release rates of 48.4% for urea and 44.46% for ammonium dihydrogen phosphate.

#### 3.3.2 Lipid-Based Systems

Lipid-based systems, including liposomes and solid lipid nanoparticles (SLNs), have also gained attention for encapsulating hydrophobic phytohormones and improving their stability and bioavailability. Liposomes are spherical vesicles composed of lipid bilayers, whereas SLNs are solid particles composed of lipids, typically with diameters ranging from 50 to 1000 nm. They consist of a solid lipid core that is stabilized by surfactants. These systems encapsulate hydrophobic phytohormones, thereby improving their stability and solubility. They can also gradually degrade to provide a sustained release. Additionally, they are helpful in delivering phytohormones that require protection from environmental factors.^95^ This unique solid lipid matrix offers advantages over traditional lipid-based carriers, such as improved stability.^96^

#### 3.3.3 Bioinspired and Biomimetic Systems

Bioinspired and biomimetic systems have emerged as promising solutions for the control of phytohormone release. These technologies mimic natural processes, such as the transport mechanisms of hormones in plants, to enhance their uptake and efficacy. Controlled-release fertilizers integrated with phytohormones represent another breakthrough that combines nutrient delivery with growth regulation. These fertilizers use polymer coatings or hybrid systems to synchronize the release of nutrients and phytohormones, thereby reducing environmental impacts while optimizing plant growth.^95,96^

#### 3.3.4 Microparticle Encapsulation

The encapsulation of microparticles involves loading active agents into a carrier matrix. This approach protects active agents against environmental factors, reduces their degradation during application, minimizes the quantity of active agents required, and allows for controlled delivery to plants. Biopolymer matrices, particularly those made from polysaccharides, such as chitosan (CS) and alginate (ALG), are highly effective for the controlled release of fertilizers.^97^ These materials can form microcapsules at ambient temperatures, making them versatile for various applications, including the immobilization and release of chemical or biologically active agents. Ionic gelation and polyelectrolyte complexation enable the simultaneous loading of essential macro- and micronutrients necessary for plant growth.^98^

In Table 5 below, emerging technologies being applied in agriculture for encapsulation and controlled release are presented. The mechanisms and the types of materials used for encapsulation are shown.

In summary, the applications of nanotechnology in agriculture currently focus on using nanomaterials for precision nutrient delivery, targeted pest control, and enhanced plant resilience, which have had considerable on-field success. Advancements include controlled-release nanofertilizers and nanopesticides that reduce waste and environmental impact, as well as nanosensors for real-time monitoring of crops and soils. However, while these innovations offer potential for improved crop productivity and sustainability, challenges related to environmental safety, toxicity, regulatory frameworks, scalability, and cost remain, which must be addressed.

The use of nanomaterials in agricultural systems involves developing and applying nanoenabled tools and technologies to improve food production, enhance crop resilience, and promote sustainability. Key advancements include the creation of nanofertilizers and nanopesticides, which enable the slow, controlled, and targeted release of nutrients and agrochemicals. These nanocarriers increase nutrient-use efficiency, reduce chemical runoff, minimize environmental contamination, and lower toxicity to nontarget organisms. Furthermore, nanomaterials enhance crop resilience against abiotic stresses, such as drought, salinity, and temperature extremes, by improving water retention and bolstering plant defense mechanisms. Nanotechnology plays a central role in the development of nanosensors and nanobiosensors for precision agriculture, enabling the real-time monitoring of soil conditions, plant health, and pathogen detection. Despite these benefits, significant challenges and risks must be addressed to ensure the safe and sustainable implementation of agricultural nanotechnology. Concerns include the potential environmental impact of nanomaterial accumulation in soil and water, which could disrupt beneficial microbial communities and affect nutrient cycles. The long-term health effects on humans and animals from exposure through the food chain or occupational contact also need further investigation. Addressing these challenges requires establishing comprehensive regulatory frameworks, developing eco-friendly synthesis methods, and conducting extensive research on the fate and effects of nanomaterials in agricultural ecosystems.

## 4 How Nanotechnology Enhances the Synergistic Interactions between Phytohormones and Microbiomes

Nanotechnology has emerged as a tool that can transform various aspects of plant growth, soil interactions, and microbial activity. By integrating nanomaterials with phytohormones, researchers aim to refine crop responses to environmental stress, optimize nutrient uptake, and enhance plant resistance to pathogens. The dynamic interaction between these technologies and the soil microbiome—a complex and diverse community of microorganisms that plays a key role in nutrient cycling, plant health, and soil fertility—further underscores the potential for synergistic benefits. Nanomaterials are being explored for their ability to interact with phytohormones at the molecular level, thereby improving their stability, bioavailability, and targeted delivery to plants. Nanotechnology can control the release of phytohormones to regulate plant growth processes, such as germination, root elongation, and flowering.^95^ Nanoparticles, such as silica, carbon-based materials, and metal oxide nanoparticles, encapsulate plant hormones, such as auxins, gibberellins, and cytokinins, ensuring more efficient and controlled release. The precise regulation of phytohormones through nanotechnology can potentially mitigate the negative impacts of abiotic stresses, such as drought, salinity, and temperature fluctuations, by optimizing plant responses at the cellular level.^99,100^

Furthermore, the interactions between nanomaterials and soil microbiomes play a significant role in enhancing plant growth. Soil microbes are instrumental in nutrient availability, disease suppression, and the promotion of plant health by modulating root architecture and facilitating symbiotic relationships.^101^ The application of nanomaterials influences the composition and activity of the soil microbiome, thereby enhancing beneficial microbial communities that support plant growth.^102^ For instance, nanoparticles can selectively promote the development of nitrogen-fixing bacteria, leading to improved nitrogen assimilation by plants or enhanced activity of phosphorus-solubilizing microbes, facilitating better phosphorus uptake.^103^ Simultaneously, concerns regarding the potential toxicity of nanoparticles to soil microbiomes have been raised because certain nanoparticles may disrupt microbial communities, particularly at high concentrations.^104,105^

The convergence of phytohormones, nanotechnology, and soil microbiomes is critical for the development of sustainable agricultural practices. Judicious application of nanomaterials in combination with phytohormones can reduce the need for synthetic fertilizers and pesticides, thereby minimizing environmental contamination and improving soil health.^42^ By enhancing natural plant resilience to stressors and promoting the efficiency of microbial processes, this integrated approach could foster a more sustainable agroecosystem in which nutrient cycling is optimized and the use of harmful chemicals is minimized. Moreover, phytohormones, such as jasmonic acid and salicylic acid, which are involved in plant defense signaling, can be modulated through nanotechnology to improve resistance against soilborne pathogens and pests, thus enhancing crop protection without relying heavily on chemical treatments.^106^ It is critical to further explore how nanomaterials and phytohormones interact with soil microbial communities at the molecular level. For instance, the impact of nanomaterials on microbial biofilms, quorum sensing, and the production of secondary metabolites, which are critical factors in plant–microbe interactions, requires further investigation.^107^ Moreover, understanding the long-term ecological implications of incorporating nanotechnology into soil systems is essential for developing best practices to ensure the sustainability of such innovations. Although nanomaterials hold great promise for agricultural advancement, their potential risks to soil health, biodiversity, and ecosystem stability must be carefully evaluated using controlled field studies and long-term monitoring.^108^

### 4.1 Modulating Plant–Microbiome Communication

Soil microorganisms are considered relevant and sensitive indicators of soil perturbations because of their key roles in biogeochemical cycling, crop production, and pollutant biodegradation.^109^ The rhizosphere, a critical interface for plant–microbe interactions, is colonized by beneficial soil-inhabiting microbes, such as bacteria and fungi, which play a pivotal role in maintaining plant and soil health.^110^ Plant–microbe symbiosis is vital for plant growth and disease protection.^111^ Therefore, a shift in soil microbial diversity can disrupt the soil ecosystem balance and compromise the ability of the soil to suppress pathogens.^112^

Nanotechnology offers a transformative approach to modulate plant-associated microbiomes, aiming to enhance plant health, growth, and stress resilience while reducing environmental harm. The integration of beneficial microorganisms and nanomaterials represents a promising strategy for improving plant health and productivity, offering a novel approach to sustainable agriculture.^113^ Over the past two decades, nanofacilitated methods have emerged as effective and sustainable alternatives to traditional microbiome engineering approaches to address challenges such as inaccurate manipulation, disruption of microbial diversity, and inconsistent outcomes.^114–116^ Nanoenabled products have unique physicochemical features, including a high surface area-to-volume ratio, greater reactivity and resilience, surface potential, modifiable physical and chemical properties, and molecular manipulation compared to their parent bulk, which can reduce reliance on synthetic fertilizers and pesticides, promoting eco-friendly and productive farming practices that benefit ecosystems and communities.^117^ Nanomaterials further enhance the activities of the soil microbiome by improving nutrient availability, water retention, and the targeted delivery of microbial inoculants to the plant root system.^118,119^ This synergistic approach enhances plant resilience to biotic and abiotic stresses,^120,121^ leading to increased crop yield and improved agricultural sustainability.^122–124^

Specifically, the interaction of microbes with nanoparticles has been shown to enhance nutrient delivery to plants, particularly under stressful conditions. The controlled release of beneficial compounds from nanomaterials further promotes plant survival.^125^ Nanoparticles also interact with rhizospheric microorganisms, thereby influencing microbial diversity and activity. These microbes produce bioactive compounds such as siderophores, lipopeptides, and exopolysaccharides, which are critical for nutrient cycling and plant health.^126^ Siderophores, for instance, chelate metals such as iron, copper, and zinc to enhance nutrient availability and reduce toxicity.^127,128^ Increased microbial siderophore synthesis accelerates the dissolution of hematite (Fe_2_O_3_) NPs, releasing iron for plant absorption.^129^ Additionally, siderophore-mobilized iron is less toxic when metabolized from iron-doped NPs with high aspect ratios.^130^ These interactions improve soil and plant health and facilitate the dissolution and aggregation of NPs with soil minerals.^131^

Xu et al.^132^ demonstrated that low doses of zinc oxide nanoparticles (ZnO NPs) significantly enhanced the plant growth-promoting potential of specific rhizospheric soil microbiota associated with lettuce, thus improving plant performance compared to untreated plants. Similarly, Mandal et al.^133^ found that zincated nanoclay polymer composites (ZNCPCs) and nanozinc oxide (Nano-ZnO) stimulate soil microbial populations, resulting in increased enzymatic activity and a higher release of DTPA-extractable zinc in the rice rhizosphere. This indicates that zinc nanoparticles may benefit plant growth-promoting rhizobacteria (PGPRs).

In addition, Chen et al.^134^ found that treating *Medicago truncatula* with metal nanoparticles significantly boosts both the diversity and abundance of soil rhizosphere microorganisms. However, it is important to note that the effects of nanoparticles were dose-dependent. Lower doses can enhance microbial metabolism and energy conversion, while higher doses may prove detrimental, as evidenced by various studies. Furthermore, the type of nanoparticles used is pivotal in determining their effects on the soil microbiota.^135,136^ Other noteworthy nanomaterials, including carbon nanotubes, sulfate-modified polystyrene nanospheres, copper oxide nanoparticles, and copper hydroxide, have also been recognized for enhancing microbial metabolism and strengthening plant–soil interactions.

Lima-Tenório et al.^137^ observed that the application of a chitosan nanomaterial-based biofertilizer containing *Azospirillum brasilense* (strains AbV5 and AbV6) to maize plants resulted in significant improvements, including a 19% increase in root length, 17% increase in shoot fresh weight, and 71% increase in chlorophyll b content. This synergistic application also extended the survival of *A. brasilense* strains in soil for at least 60 days.

In addition, chitosan-immobilized silica nanocomposites (CISNC) containing *Glomus mosseae*, *Trichoderma viridae*, and *Bacillus subtilis* were found to be effective in mitigating tomato bacterial wilt caused by *Ralstonia solanacearum*.^138^ These nanocomposites also improve water retention and enhance physiological, biochemical, and soil microbial activities, contributing to an increased tomato yield and resource-use efficiency.^139^ Similarly, nanobiofertilizers composed of nanoclay-encapsulated *Trichoderma* and *Pseudomonas* species have induced resistance to fungal and nematode diseases, as well as other abiotic stress factors in Rabi crops.^43^ The combined action of beneficial microbes and nanomaterials enhances nutrient uptake and utilization, strengthens plant defense mechanisms, and reduces stress-induced oxidative damage. Figures 5 and 6 below show how nanoagrochemicals can be synergistically applied with rhizobacteria to influence the performance of plants and enhance their resistance to biotic and abiotic stress. This multifaceted approach promises to revolutionize sustainable agriculture by promoting plant health and productivity while minimizing environmental impacts. However, there is a need to optimize the combination of specific microbial consortia and nanomaterials tailored to different plant species and environmental conditions to maximize synergistic effects and achieve sustainable intensification of agricultural practices.

**Figure 5:**
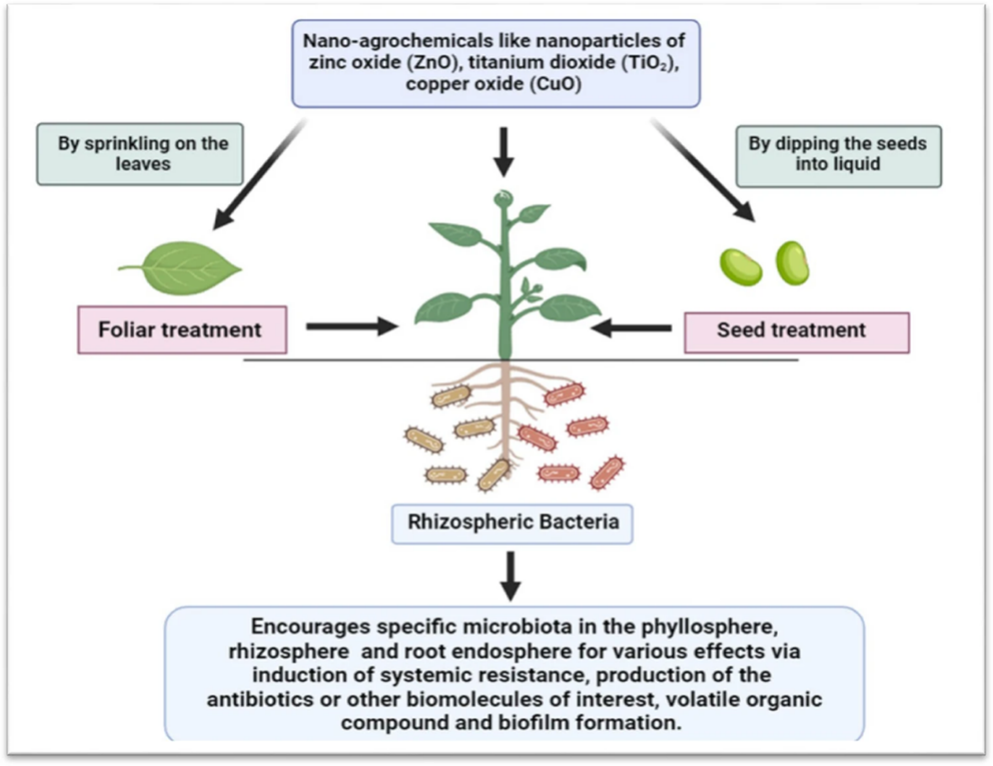
Role of nanoagrochemicals in inducing different mechanisms. Reprinted with permission from Meel, S. and Saharan, B.S. (2024). Enhancing crop resilience toward drought: by integrating nanotechnology, microbiomes, and growth-promoting rhizobacteria. *Discov Agric*, 2, 112. Copyright 2024/Springer Nature.

**Figure 6:**
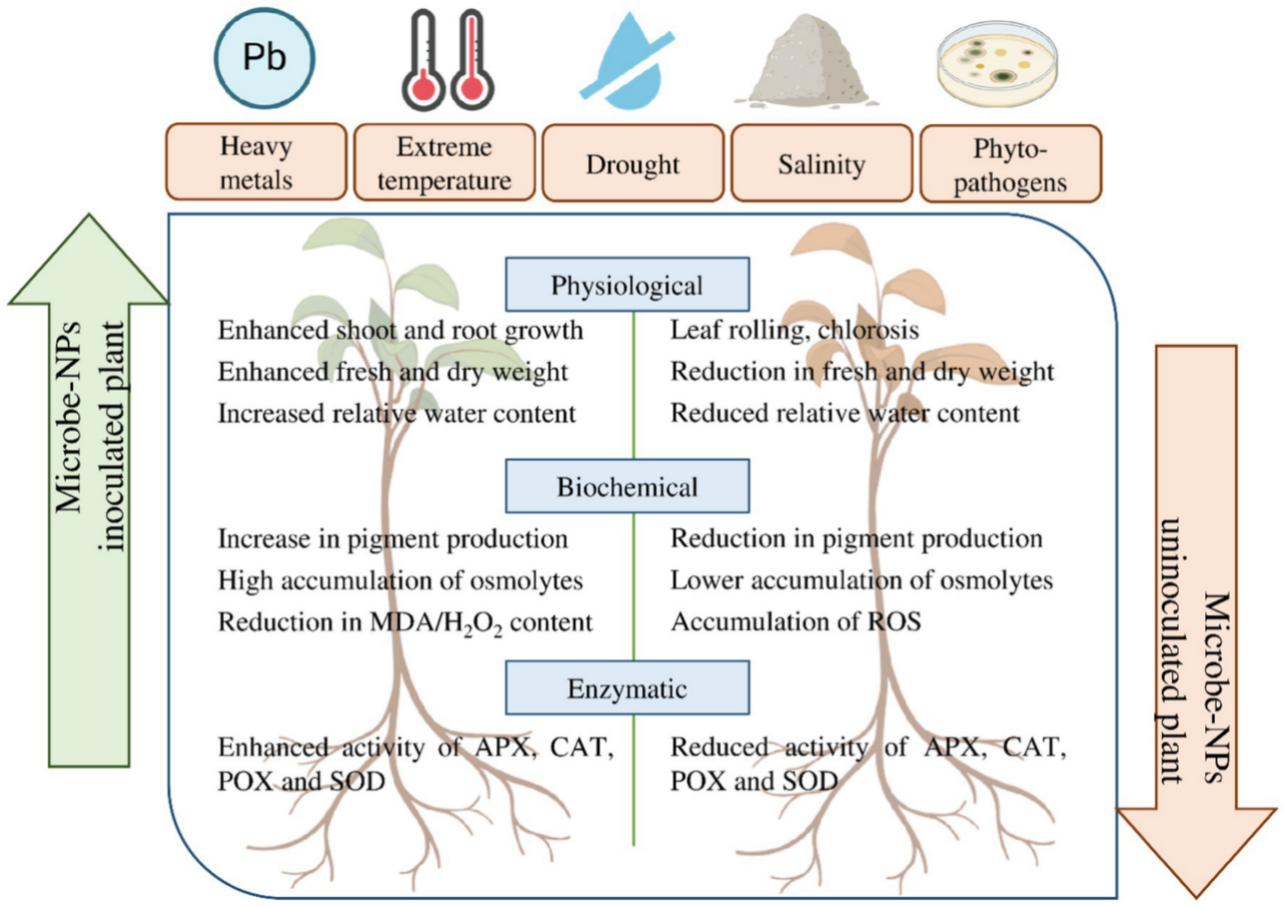
Synergistic effects of microbe–nanoparticles on physiological, biochemical, and enzymatic attributes of plants under biotic and abiotic stresses. Reprinted with permission from Sodhi, G. K., Wijesekara, T., Kumawat, K. C., Adhikari, P., Joshi, K., Singh, S., Farda, B., Djebaili, R., Sabbi, E., Ramila, F., Sillu, D., Santoyo, G., Kumar, A., Pellegrini, M., & Mitra, D. (2025). Nanomaterials–plants–microbes’ interaction: Plant growth promotion and stress mitigation. *Frontiers in Microbiology*, 15, 1516794. Copyright 2025/Frontiers.

## 5 How Nanophyto–Micro Systems Address Agricultural Challenges

Climate change and rising populations have significantly affected food security.^140^ Abiotic stress harms major crops worldwide by affecting photosynthesis, respiration, growth, and development. Stresses, such as drought, salinity, heavy metals, extreme temperatures, and submergence, affect various growth stages, leading to a loss of crop productivity.^141^ Agriculture is pivotal in ensuring food security because the human population relies heavily on crop-based foods for essential nutrients.^142^ If global warming is not adequately addressed, then various abiotic stresses may affect more arable land in the coming decades.^143^

To counter these challenges, climate-smart agriculture is emerging as a strategic approach to mitigate the adverse effects of environmental stress on crops. The conventional use of agrochemicals to manage these stressors raises concerns regarding ecological contamination and poses significant risks to human health.^144^ Plant probiotics have gained attention as viable alternatives because of their potential role in alleviating both abiotic and biotic stresses in crops.^145^ Integrating nanoagrochemicals into beneficial plant microbiomes is a promising strategy for enhancing plant resilience to biotic and abiotic stresses by activating specific morphophysiological and genetic mechanisms. The plant microbiome plays a crucial role in promoting growth and mitigating stress through various mechanisms, such as improving nutrient acquisition and water availability, maintaining oxidative homeostasis, and synthesizing phytohormones that enhance plant fitness under stress.^146,147^

Recent studies have indicated that the synergistic use of PGPR and NPs is more effective in boosting crop productivity than their individual application.^148–150^ For instance, combining *Alcaligenes* spp. with NPs enhances fresh and dry biomass, plant height, yield, and fruit quality, while reducing fertilizer usage and greenhouse gas emissions from fertilizer production.^148,151,152^ Recent advancements in the engineering of plant-associated microbiomes, primarily through nanoenabled methods, have demonstrated significant potential over traditional techniques by providing specificity and minimizing collateral damage to microbial diversity. The goal is to create a sustainable agricultural framework that enhances crop health and productivity while reducing the reliance on synthetic fertilizers and pesticides, thereby lessening the negative impacts of agriculture on the environment and human health.^137^ Empirical studies have indicated that inorganic nanomaterials can alter the plant microbiome composition. For instance, iron-based nanoparticles significantly increase the number of beneficial microbial groups associated with soybeans.^153^ Carbon-based engineered nanomaterials have also shown promise in modulating plant-associated microbiomes; amendments with multiwalled carbon nanotubes enhanced beneficial microbial communities in black nightshade.^37^ Polymeric ENMs, such as chitosan, have improved the rhizosphere microbiome in maize.^154^ Nanocomposites have gained attention owing to their role in enhancing agricultural efficacy by stabilizing and modulating microbial communities.

### 5.1 Applications of Nanotechnology with Phytohormone–Microbiome Systems for Sustainable Agriculture

#### 5.1.1 Stress Management

Nanotechnology enhances abiotic stress tolerance in plants by delivering protective agents such as antioxidants, osmoprotectants, and signaling molecules, which mitigate stress-induced damage. Nanomaterials, such as silica and carbon-based nanoparticles, have been found to promote more profound and extensive root systems that enhance resource acquisition, branching, and density by delivering phytohormones such as auxins and cytokinins, which aid in improving water and nutrient uptake under stressful conditions.^155,156^ In addition to root architecture, nanotechnology influences microbial activity in the rhizosphere by protecting and delivering beneficial microbes such as nitrogen-fixing bacteria and phosphate-solubilizing microorganisms.^45^ Encapsulated microbes are shielded from environmental stresses, enhancing their survival and activity, which improves nutrient cycling and plant–microbe interactions. This synergy between nanomaterials, root architecture, and microbial activity helps plants better tolerate abiotic stresses, such as drought, salinity, and nutrient deficiencies.^87^ Recent studies have established that the potential synergistic effects of combining plant probiotics with nanomaterials may lead to substantial improvements in managing abiotic stress.^157–162^

For example, *Bacillus pumilus* combined with silver nanoparticles (Ag-NPs) improved the growth of onions under saline conditions, enhancing moisture retention and bulb protein content.^163^ Furthermore, NPs can alleviate drought stress and reduce soil contaminant phytotoxicity by improving soil water retention and decreasing sodium absorption.^119,164–167^ The effectiveness of plant probiotics stems from their inherent tolerance to various stressors, which is conferred by multiple mechanisms, including the production of exopolysaccharides, accumulation of osmoprotectants, production of ACC deaminase, and activation of stress-responsive genes.^168^

This combination enhances stress amelioration by improving the levels of photosynthetic pigments, the accumulation of osmolytes, and phenolic compound activities of antioxidant enzymes and total soluble sugars, while simultaneously reducing stress markers such as malondialdehyde and electrolyte leakage in plants subjected to salt and drought stress.^158,169,170^ This integrated approach holds promise for enhancing crop resilience and ensuring sustainable agricultural practices in the face of global climate change (Figure 7).

**Figure 7:**
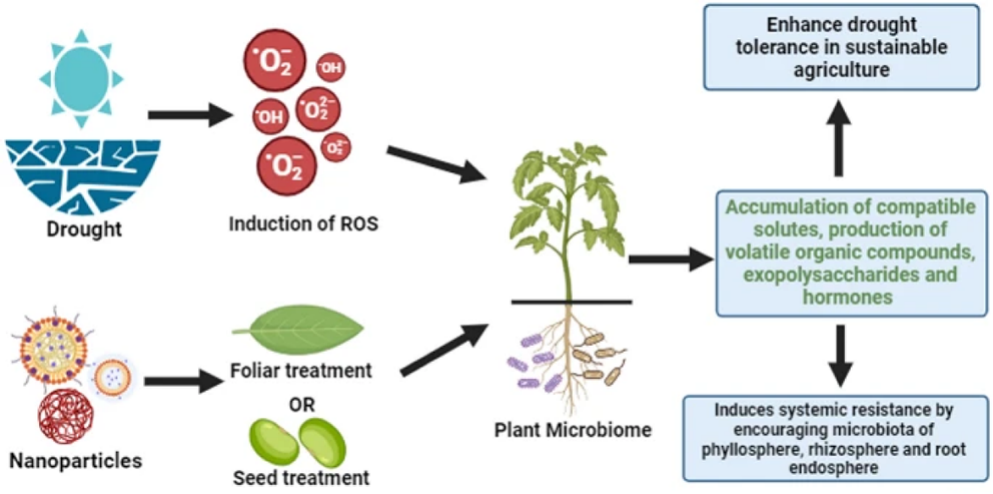
Synergistic role of nanotechnology, plant microbiome, and phytohormones in enhancing drought tolerance in sustainable agriculture. This diagram illustrates how drought stress leads to the accumulation of reactive oxygen species (ROS), negatively affecting plant health. Applying nanoparticles via foliar spray or seed treatment can modulate the plant microbiome, enhancing its capacity to respond to stress. The microbiome, in turn, promotes the accumulation of compatible solutes, volatile organic compounds, exopolysaccharides, and phytohormones—key components in mitigating drought effects. This interaction induces systemic resistance by stimulating beneficial microbial communities in the phyllosphere, rhizosphere, and root endosphere, ultimately contributing to improved plant resilience and productivity under water-limited conditions. Reprinted with permission from Meel, S. and Saharan, B.S. (2024). Enhancing crop resilience toward drought: by integrating nanotechnology, microbiomes, and growth-promoting rhizobacteria. *Discov Agric*, 2, 112. Copyright 2024/Springer Nature.

#### 5.1.2 Nutrient Use Efficiency

Nanomaterials enhance nutrient uptake efficiency by improving nutrient bioavailability, enabling controlled release, and protecting beneficial microbes, such as nitrogen-fixing bacteria, from environmental stresses.^45^ They also deliver growth-promoting hormones such as auxins to stimulate root development and increase the root surface area for nutrient absorption. Additionally, these systems minimize nutrient losses through targeted delivery, reduce the need for excessive chemical fertilizers, and mitigate environmental pollution.^171^ Various PGPR, such as *Enterobacter*, *Pseudomonas*, *Azospirillum*, and *Agrobacterium*, paired with NPs, significantly improve seed germination, plant height, and biomass.^172–174^

PGPR also benefit plants by producing phytohormones and enhancing nutrient availability, while NPs provide a nutrient-rich substrate that increases PGPR effectiveness.^148,149,162,172^ Studies combining nanomaterials with plant growth-promoting bacteria (PGPBs) have demonstrated that zinc oxide NPs and PGPBs can reduce DNA damage and cytosine methylation levels, thereby enhancing stress resilience in tomato seedlings.^173^ In addition, selenium nanoparticles increased crop yield and photosynthetic efficiency in pakchoi,^153^ whereas copper oxide nanoparticles reshaped the nitrogen cycle in wheat.^174^ In summary, the combined application of nanoagrochemicals and beneficial microbiomes offers an innovative approach to sustainable agriculture.

#### 5.1.3 Disease Resistance

Nanoparticles encapsulating salicylic acid and biocontrol agents enhance plant immunity and suppress pathogens.^166^ These nanoparticles offer a cutting-edge approach for improving plant immunity and suppressing pathogens. Salicylic acid is a key signaling molecule in plant defense that activates systemic acquired resistance (SAR) and produces pathogenesis-related proteins.^175^ However, its practical application is limited by rapid degradation and nontargeted delivery. Encapsulating SA in nanoparticles overcomes these challenges by protecting them from environmental degradation, enabling controlled release and ensuring targeted delivery to specific plant tissues.^176^

Similarly, biocontrol agents, such as beneficial microbes such as *Trichoderma* and *Pseudomonas fluorescens*, often face environmental stresses that reduce their efficacy. Nanoparticle encapsulation protects these microbes from adverse conditions by enhancing their survival, colonization, and ability to antagonize pathogens.^177^ Combining SA and biocontrol agents within nanoparticle systems creates a synergistic effect, providing dual action against pathogens. SA primes the plant immune system, whereas biocontrol agents suppress pathogens through competition, antimicrobial compound production, and the induction of systemic resistance.^104^ Nanoparticle systems, such as chitosan, silica, and biodegradable polymers, enable sustained release and targeted delivery, enhancing the stability and efficiency of both components.^171^ This technology has shown promise in reducing plant diseases, improving stress tolerance, and increasing crop yields, thus making it a sustainable solution for modern agriculture. However, environmental safety, cost, and scalability must be addressed to ensure its widespread adoption and long-term benefits.^46^

In conclusion, nanophyto–micro systems offer a holistic and sustainable approach to addressing major agricultural challenges, leveraging the combined power of nanotechnology, beneficial microorganisms, and natural plant compounds. These integrated platforms enhance crop productivity, fortify plants against pests and diseases, and improve resilience to environmental stresses such as drought and salinity. By working in synergy, the components deliver targeted and highly efficient solutions that minimize the ecological damage often associated with conventional farming practices.

One key area where this technology provides significant benefits is in optimizing plant growth and nutrient delivery. Nanofertilizers can encapsulate essential nutrients and release them in a slow, controlled manner directly to the plant’s roots, dramatically improving nutrient-use efficiency and reducing fertilizer runoff. Beneficial soil microorganisms are integrated into this process, aiding in nutrient cycling and enhancing the plant’s uptake of vital resources. This precision delivery minimizes waste and optimizes plant development by ensuring a steady supply of nutrients, leading to increased yields and healthier crops. Furthermore, nanophyto–micro systems provide powerful defenses against pathogens and environmental pressures. Nanopesticides and natural antimicrobial agents can target harmful organisms with high specificity while using lower concentrations of chemicals, thereby reducing toxicity to nontarget species and the broader environment. Simultaneously, the system strengthens the plant’s natural immune responses using phytonutrients and beneficial microbes, which produce antifungal and antibacterial compounds. This multipronged strategy boosts the plant’s resilience, enabling it to better withstand attacks from pests and tolerate adverse conditions, such as drought, extreme temperatures, and soil salinity.

## 6 Translational Pathways and Real-World Implementation

As discussed in this manuscript, applying nanotechnology in conjunction with phytohormones and beneficial soil microorganisms is a promising approach for promoting plant growth, improving resource use efficiency, and increasing crop resilience to abiotic stress. However, despite these positive aspects, significant challenges remain to ensure that farmers fully adopt these technologies.^178^

One of the main challenges in nanotechnology is scalability. The key objective of these processes is to carry out large-scale production while maintaining the same physical and chemical characteristics.^179^ Additionally, high investments in infrastructure, materials, and quality control often affect the final cost of products and their adoption by farmers. Despite their superior performance, many of these nanotechnology-based solutions still struggle to be competitive with conventional products.^180,181^ In this context, for large-scale adoption, these products must also be compatible with traditional agricultural application methods, such as sprayers and seed treatments.^182^

Regulatory frameworks are another factor that significantly affects the adoption of these technologies.^183^ Several countries do not have specific guidelines for nanotechnology-based agricultural inputs, creating uncertainty for production companies and farmers. Some products may even be classified into existing categories. However, not considering their unique characteristics can make it difficult for them to enter the market.^184^ In this context, it is also necessary for these regulatory frameworks to consider the physical–chemical, toxicological, and environmental aspects of these formulations.^185^

To reach farmers, these formulations must undergo different validation phases, including tests in real environments such as in the field. In this sense, some studies already published in the literature have demonstrated the effectiveness of these nanotechnology-based systems under real cultivation conditions, reinforcing their potential for practical applications. Pereira et al.^186^ investigated, under field conditions, the use of polymeric nanoparticles containing gibberellic acid (GA_3_) for treating tomato (*Solanum lycopersicum*) seeds and showed that using nanoparticles, especially those based on alginate/chitosan, significantly increased plant productivity and almost quadrupled fruit production compared with the control. Farhangi-Abriz et al.^187^ also evaluated the effects of foliar application of carbon quantum dots (CQDs) on soybean plants subjected to water stress under field conditions. The results showed that, under water stress, CQDs significantly improved several physiological parameters, including green soil cover (approximately 14%), leaf area (21%), chlorophyll content (18%), maximum efficiency of photosystem II (19%), and the relative electron transport rate in photosynthesis (23%). In addition, CQDs increased the leaf water content, osmolyte production, antioxidant activity, and grain yield (25%).

Finally, for these innovations to reach the field and affect the lives of rural producers and consumers, it is necessary to involve several actors throughout the production chain. This goes beyond the farmer and includes formulating companies, asset companies, research centers, universities, regulatory agencies, and public policymakers.^188^ The connection between science, industry, and society is a key factor for overcoming technical challenges, validating benefits in the field, and transforming innovations into real solutions for agriculture.^189^

## 7 Conclusions

The integration of nanotechnology, plant-associated microbiomes, and phytohormone signaling represents a transformative opportunity for next-generation agricultural systems. Collectively, these technologies have enabled the development of innovative and responsive platforms for targeted delivery, enhanced plant resilience, and sustainable crop protection. Our perspective highlights how this synergistic approach can overcome the current limitations of conventional agriculture by improving bioavailability, reducing environmental toxicity, and aligning itself with regenerative practices. Robust interdisciplinary collaboration between materials scientists, plant biologists, microbiologists, and agricultural engineers is required to translate this vision into practical solutions. Furthermore, advancing these technologies from bench to field requires supportive policy frameworks and industry engagement to enable scalable and cost-effective implementation. By synthesizing these frontier technologies, we can catalyze the development of resilient, eco-conscious, and data-driven agricultural systems. This integrated strategy addresses urgent global challenges, such as food security and climate resilience, and sets the foundation for a new paradigm in sustainable agriculture.
